# Respondent-driven sampling to assess mental health outcomes, stigma and acceptance among women raising children born from sexual violence-related pregnancies in eastern Democratic Republic of Congo

**DOI:** 10.1136/bmjopen-2014-007057

**Published:** 2015-04-08

**Authors:** Jennifer Scott, Shada Rouhani, Ashley Greiner, Katherine Albutt, Philipp Kuwert, Michele R Hacker, Michael VanRooyen, Susan Bartels

**Affiliations:** 1 Department of Obstetrics and Gynecology, Beth Israel Deaconess Medical Center, Boston, Massachusetts, USA; 2 Division of Women's Health, Brigham and Women's Hospital, Boston, Massachusetts, USA; 3 Harvard Humanitarian Initiative, Cambridge, Massachusetts, USA; 4 Harvard Medical School, Boston, Massachusetts, USA; 5 Department of Emergency Medicine, Brigham and Women's Hospital, Boston, Massachusetts, USA; 6 Beth Israel Deaconess Medical Center, Department of Emergency Medicine, Boston, Massachusetts, USA; 7 Department of General Surgery, Massachusetts General Hospital, Boston, Massachusetts, USA; 8 Department of Psychiatry and Psychotherapy, University Medicine Greifswald, HELIOS Hansehospital Stralsund, Stralsund, Germany; 9 Department of Epidemiology, Harvard School of Public Health, Boston, Massachusetts, USA; 10 Harvard School of Public Health, Boston, Massachusetts, USA

**Keywords:** MENTAL HEALTH, PUBLIC HEALTH, OBSTETRICS

## Abstract

**Objectives:**

Assess mental health outcomes among women raising children from sexual violence-related pregnancies (SVRPs) in eastern Democratic Republic of Congo and stigma toward and acceptance of women and their children.

**Design:**

Participants were recruited using respondent-driven sampling.

**Setting:**

Bukavu, Democratic Republic of Congo in 2012.

**Participants:**

757 adult women raising children from SVRPs were interviewed. A woman aged 18 and older was eligible for the study if she self-identified as a sexual violence survivor since the start of the conflict (∼1996), conceived an SVRP, delivered a liveborn child and was currently raising the child. A woman was ineligible for the study if the SVRP ended with a spontaneous abortion or fetal demise or the child was not currently living or in the care of the biological mother.

**Intervention:**

Trained female Congolese interviewers verbally administered a quantitative survey after obtaining verbal informed consent.

**Outcome measures:**

Symptom criteria for major depressive disorder, post-traumatic stress disorder, anxiety and suicidality were assessed, as well as stigma toward the woman and her child. Acceptance of the woman and child from the spouse, family and community were analysed.

**Results:**

48.6% met symptom criteria for major depressive disorder, 57.9% for post-traumatic stress disorder, 43.3% for anxiety and 34.2% reported suicidality. Women who reported stigma from the community (38.4%) or who reported stigma toward the child from the spouse (42.9%), family (31.8%) or community (38.1%) were significantly more likely to meet symptom criteria for most mental health disorders. Although not statistically significant, participants who reported acceptance and acceptance of their children from the spouse, family and community were less likely to meet symptom criteria.

**Conclusions:**

Women raising children from SVRPs experience symptoms of mental health disorders. Programming addressing stigma and acceptance following sexual violence may improve mental health outcomes in this population.

**Strengths and limitations of this study**

- The study provides data on mental health outcomes, stigma and acceptance among a population of women that has not been included in other research approaches.
- The sample was large, and in this study, respondent-driven sampling may approximate a random sample.
- We relied on self reporting; however, with the screening questions, sensitive nature of sexual violence-related pregnancies (SVRPs) and minimal incentive, we are confident that only eligible participants were interviewed.
- Further, mental health disorders were classified based on symptom criteria and diagnostic interviews were not conducted. It is possible that questions were misinterpreted or the instruments do not match local constructs of mental health outcomes, stigma and acceptance.
- Owing to regional insecurity, the study was terminated early; thus, potential participants with coupons may not have been interviewed. Despite the early study termination, over 750 women presented for interviews within a 4-week study period.

## Background

Sexual violence, widespread in eastern Democratic Republic of Congo (DRC),^1^ ^2^ has mental health consequences, including depression, post-traumatic stress disorder (PTSD), anxiety and suicidal ideation.^1^ ^3^ ^4^ In DRC, stigma and social rejection as a result of sexual violence have been reported^1^ ^5–10^ and having a child from sexual violence is a risk factor for social rejection.^5^ Data on sexual violence-related pregnancies (SVRPs) are limited, but studies in DRC estimated the prevalence of SVRPs to be 6–17% among sexual violence survivors.^1^ ^5^ ^11^

Reports from DRC and other conflict settings suggest that having a child from an SVRP leads to complex psychosocial phenomena, including stigma,^8^ ^12–14^ but there are limited data on mental health outcomes among women who experienced SVRPs. Several studies indicate that stigmatisation plays a mediating role in the relationship between sexual violence and mental health outcomes in conflict settings,^9^ ^15–17^ but these studies have not focused on women with SVRPs. There is increasing attention on mental health programming for sexual violence survivors in conflict settings,^3^ ^18^ ^19^ and data on SVRPs could further inform programming.

This study primarily aimed to assess mental health outcomes among women raising children from SVRPs in eastern DRC. The study also aimed to determine: (1) perceived stigma toward women by the community and stigma toward the child from the woman's spouse, family, and community and (2) perceived acceptance of the woman and her child from the spouse, family and community.

## Methods

The study was conducted in Bukavu, South Kivu Province, DRC, in October–November 2012.

### Participant recruitment

Study participants were recruited using respondent-driven sampling (RDS)—a peer-to-peer recruitment system used to sample hard-to-reach populations.^20^ ^21^ The details of RDS methodology for this study were previously described.^22^

There were two study groups: (1) women raising children from SVRPs (parenting group) and (2) women who terminated SVRPs (termination group). Only parenting group data are presented in this manuscript. A woman aged 18 and older was eligible for the study if she self-identified as a sexual violence survivor since the start of the conflict (∼1996), conceived an SVRP, delivered a liveborn child and was currently raising the child. A woman was ineligible for the study if the SVRP ended with a spontaneous abortion or fetal demise, or the child was not currently living or in the care of the biological mother.

### Procedure

Partner organisations identified 18 initial participants who met eligibility criteria and were well networked in the community (8 for the parenting group, 8 for the termination group and 2 were eligible for both groups). The initial participants were not included in the analysis. Each initial participant received three uniquely numbered coupons with a 2-week expiration date to distribute to three other potentially eligible women. The women received verbal instructions about the study purpose and were asked to recruit participants for both study groups.

On arrival to the study centre with a valid coupon, a recruited participant answered standardised screening questions to confirm study eligibility. Following verbal informed consent, trained female Congolese interviewers verbally administered the survey in a private setting. On completion, participants received an incentive (a headscarf), transport reimbursement and three coupons to distribute to three potentially eligible women. A secondary incentive for recruitment was not provided. Recruitment was tracked through coupon numbering. Details on recruitment patterns were previously described.^22^ Owing to regional insecurity, the study investigators terminated the study early after a 4-week period of data collection.

### Instruments

The survey was developed in collaboration with partner organisations and field experts, written in English, translated into Kiswahili and back translated. A panel resolved translation discrepancies. Cognitive interviewing and pilot testing of survey instruments were conducted prior to survey administration. The survey was verbally administered in Kiswahili and the interviewer recorded responses on electronic handheld devices using KOBO technology.^23^

Mental health outcomes were assessed with instruments previously used in conflict settings and to assess survivors of violence.^1^ ^3^ ^24^ ^25^ Using a cut-off score of ≥3, validated short forms of the Patient Health Questionnaire PHQ-9 (PHQ-2)^26^ and the Generalised Anxiety Disorder GAD-7 (GAD-2)^27^ assessed symptoms of depression and anxiety, respectively. Using a cut-off score of ≥50, the PTSD Checklist-Civilian Version (PCL-C) assessed symptoms of PTSD.^28^ Women were asked about suicidal ideation and suicide attempts. Owing to a programming error, 74 participants who reported ‘no’ to the first GAD-2 question were not asked the second question and were not considered to meet criteria for anxiety.

The 99-question survey instrument included five questions on stigma and six questions on acceptance. Perceived stigma toward the woman by the community and stigma toward the child from the woman's spouse, family and community were assessed using five-point Likert scale questions (ie, “I believe that the community stigmatises me because I have a child born from sexual violence”). Perceived acceptance of the woman and her child from the spouse, family and community were assessed with five-point Likert scale questions (ie, “My family members accept my child as he/she is”).

### Definitions

Sexual violence was defined as “intercourse against your will, being forced to undress, molestation, and other unwanted sexual acts”. Gang rape was defined as two or more perpetrators. Sexual captivity was defined as >24 h in captivity of a sexual nature. An SVRP was any pregnancy self-reported to have been conceived as a result of sexual violence. Perpetrator(s) refer to the person(s) who inflicted sexual violence. If participants reported raising more than one child from sexual violence, data from the oldest child and from that episode of sexual violence were analysed.

### Ethical Approval

Ethical approval was obtained from Harvard School of Public Health, the Provincial Minister of Health and Medical Inspector, and the study's community advisory board. Two trained psychosocial assistants offered on-site counselling. All participants received a referral card for medical and/or mental healthcare. Interviewers completed a 6-day training and had prior research experience and/or experience working with sexual violence survivors. Identifying information was not collected and study-related documents did not disclose the nature of the study. Electronic devices and data were password protected and files encrypted.

### Statistical Analysis

Analysis of recruitment patterns and potential biases was conducted using RDSAT 7.1.38.^29^ RDSAT population proportion weighting estimates were generated from the mean network size for all variables. A variable was weighted for in the final analysis if it had a homophily of >0.3 or if its population proportion estimate required over a 5% correction. Of nine variables analysed, five (religion, ethnicity, place of origin, current residency and marital status) met these criteria and required weighting. Cross recruitment between the study groups prevented analysis of recruitment patterns by individual group; thus, weights to account for over-recruitment or under-recruitment were generated using the entire study population.^22^ All further data analysis was performed using SAS V.9.3.^30^ Likert scale responses “strongly agree” or “agree” were considered affirmative. Suicidal ideation or attempt was analysed as “suicidality”. Data are reported as mean±SD or proportion. A χ^2^ test was used for comparisons and Poisson regression was used to calculate risk ratios and 95% CIs for crude and multivariable models. Variables significant on univariate testing and known confounders were included in the multivariable model and are listed in tables 4 and 5. All tests were two-sided and p<0.05 was considered statistically significant.

## Results

Among the 18 initial participants for the study, 12 recruited respondents and 6 did not recruit.^22^ Among respondents interviewed, 53.3% made at least one referral and 46.7% did not recruit any participants.^22^ In total, 757 women were interviewed for the parenting group.

Participants were 33.8±9.1 years of age and the children were 5.7±2.3 years of age. The majority resided in Bukavu (76.1%), reported Bashi ethnicity (74.0%), and were Catholic (53.2%) or Protestant (45.1%). At the time of the survey, women reported being married (31.9%), divorced or separated (28.7%), widowed (20.5%) or never married (12.2%); the remaining (6.8%) reported husband missing, living with partner, or other. The majority (88.5%) reported one sexual violence incident, while 11.5% reported more than one incident. The most common dates of sexual violence were 2004–2008. The majority of women reported ≥2 perpetrators (80.6%) and that the pregnancy was conceived in captivity (85.5%). Over 90% reported that the perpetrators were from a single armed group.

Among participants, 48.6% met symptom criteria for depression, 57.9% for PTSD, and 43.3% for anxiety; 34.2% reported suicidality. Women who reported having a spouse at the time of the survey were less likely to meet criteria for depression (32.8% vs 56.3%, p<0.0001), PTSD (50.6% vs 61.4%, p=0.004), anxiety (33.4% vs 47.7%, p=0.0002) and suicidality (19.1% vs 40.9%, p<0.0001) compared to women who did not report having a spouse (table 1). Women who reported ≥2 perpetrators were more likely than those who reported 1 perpetrator to meet symptom criteria for PTSD (59.7% vs 49.3%, p=0.02) and suicidality (36.0% vs 26.5%, p=0.03). There were no differences in mental health outcomes among women whose SVRP resulted from captivity compared to those who did not report captivity.

**Table 1:** Mental health outcomes stratified by demographic characteristics and sexual violence incident characteristics

	Depression			PTSD			Anxiety			Suicidality*		
Variable	Yes	No	p Value	Yes	No	p Value	Yes	No	p Value	Yes	No	p Value
*Demographic characteristics*												
Has a spouse			<0.0001			0.004			0.0002			<0.0001
Yes	82 (32.8%)	168 (67.2%)		126 (50.6%)	123 (49.4%)		83 (33.4%)	166 (66.6%)		48 (19.1%)	202 (80.9%)	
No	289 (56.3%)	224 (43.7%)		315 (61.4%)	198 (38.6%)		244 (47.7%)	268 (52.3%)		210 (40.9%)	303 (59.1%)	
Education			0.006			0.84			0.005			0.009
Any education	222 (53.1%)	196 (46.9%)		243 (58.2%)	175 (41.8%)		200 (47.9%)	217 (52.1%)		159 (38.1%)	259 (61.9%)	
No education	148 (42.4%)	197 (57.1%)		198 (57.5%)	147 (42.5%)		130 (37.9%)	213 (62.1%)		100 (28.9%)	245 (71.1%)	
*Sexual violence incident characteristics*												
Number of perpetrators			0.25			0.02			0.13			0.03
Two or more	298 (49.2%)	308 (50.8%)		361 (59.7%)	244 (40.3%)		269 (44.5%)	336 (55.6%)		218 (36.0%)	388 (64.1%)	
One	64 (43.9%)	82 (56.1%)		72 (49.3%)	74 (50.7%)		55 (37.5%)	91 (62.5%)		39 (26.5%)	107 (73.5%)	
Pregnancy from sexual captivity			0.48			0.66			0.96			0.20
Yes	308 (48.5%)	327 (51.5%)		368 (57.9%)	267 (42.1%)		271 (42.7%)	364 (57.3%)		218 (34.4%)	417 (65.6%)	
No	48 (44.8%)	59 (55.2%)		60 (55.6%)	48 (44.4%)		46 (43.0%)	61 (57.1%)		30 (28.1%)	77 (71.9%)	
Remembers perpetrator when sees the child			0.56			<0.0001			0.01			0.77
Yes	250 (49.4%)	256 (50.6%)		326 (64.4%)	180 (35.6%)		235 (46.5%)	270 (53.5%)		174 (34.4%)	332 (65.7%)	
No	123 (47.2%)	137 (52.8%)		117 (45.0%)	143 (55.0%)		96 (37.1%)	164 (62.9%)		86 (33.3%)	173 (66.7%)	

*Suicidality includes women who reported suicide ideation or suicide attempt.

SVRP, sexual violence-related pregnancy.

Stigma toward the woman from the community was reported by 38.4% of participants. Stigma was reported in varied ways: “I have been emotionally abused” (26%), “they make me feel dirty” (24.5%), “I have been raped” (23.7%), “I am not accepted by my peers” (20.6%), and “I am not allowed to participate in social events” (7.9%); however, 36.2% of participants reported no stigma. Women who reported “the community stigmatizes me” were more likely to meet criteria for depression (56.8% vs 43.9%, p=0.002), PTSD (71.9% vs 52.0%, p<0.0001), anxiety (60.5% vs 35.8%, p<0.0001), and suicidality (47.1% vs 25.7%, p<0.0001; table 2).

**Table 2:** Reported stigma and mental health outcomes among women raising children born from SVRPs

	Depression			PTSD			Anxiety			Suicidality*		
Variable	Yes	No	p Value	Yes	No	p Value	Yes	No	p Value	Yes	No	p Value
The community stigmatises me†			0.002			<0.0001			<0.0001			<0.0001
Yes	133 (56.8%)	101 (43.2%)		169 (71.9%)	66 (28.1%)		142 (60.5%)	93 (39.5%)		110 (47.1%)	124 (52.9%)	
No	165 (43.9%)	211 (56.1%)		195 (52.0%)	180 (48.0%)		134 (35.8%)	241 (64.3%)		96 (25.7%)	279 (74.3%)	
Stigma toward child from spouse, family, or community			<0.0001			<0.0001			<0.0001			<0.0001
Yes	216 (55.9%)	170 (44.1%)		269 (70.0%)	117 (30.4%)		206 (53.4%)	180 (46.6%)		161 (41.7%)	225 (58.3%)	
No	157 (41.3%)	223 (58.7%)		174 (45.9%)	205 (54.1%)		125 (33.0%)	254 (67.0%)		99 (26.2%)	280 (73.8%)	
Stigma toward child from spouse‡			<0.0001			0.0002			0.002			0.01
Yes	78 (54.7%)	64 (45.3%)		96 (67.9%)	46 (32.1%)		73 (51.5%)	69 (48.5%)		52 (37.0%)	89 (63.0%)	
No	56 (29.8%)	133 (70.2%)		90 (47.7%)	99 (52.3%)		65 (34.2%)	124 (65.8%)		45 (23.9%)	144 (76.1%)	
Stigma toward child from family§			<0.0001			<0.0001			<0.0001			<0.0001
Yes	141 (59.3%)	97 (40.7%)		171 (72.1%)	66 (27.9%)		138 (57.9%)	100 (42.1%)		111 (46.6%)	127 (53.5%)	
No	219 (43.0%)	291 (57.0%)		261 (51.1%)	250 (48.9%)		185 (36.4%)	325 (63.7%)		141 (27.6%)	369 (72.4%)	
Stigma toward child from community†			0.02			<0.0001			0.0004			0.003
Yes	128 (55.1%)	104 (44.9%)		168 (72.2%)	65 (27.8%)		129 (55.3%)	104 (44.7%)		97 (41.7%)	136 (58.3%)	
No	171 (45.3%)	206 (54.7%)		196 (52.1%)	181 (47.9%)		153 (40.6%)	224 (59.4%)		112 (29.7%)	265 (70.3%)	

*Suicidality includes women who reported suicide ideation or suicide attempt.

†Those who reported that the community was not aware of the sexual violence were excluded from the analysis.

‡Those who did not report a spouse or who reported their spouse did not know of the sexual violence were excluded from the analysis.

§Those who reported their family was not aware of the sexual violence were excluded from this analysis.

SVRP, sexual violence-related pregnancy.

Participants who reported stigma toward the child from the spouse (42.9%), family (31.8%), and the community (38.1%) were more likely to meet criteria for all mental health outcomes (all p<0.05). Women who reported acceptance following sexual violence and/or acceptance of the child from the spouse, family and /or community following sexual violence were less likely to meet criteria for mental health disorders (table 3).

**Table 3:** Reported acceptance and mental health outcomes among women raising a child born from a SVRP

	Depression			PTSD			Anxiety			Suicidality*		
Variable	Yes	No	p Value	Yes	No	p Value	Yes	No	p Value	Yes	No	p Value
Spouse, family or community accepts me after SV†			<0.0001			<0.0001			0.0002			<0.0001
Yes	263 (43.9%)	335 (56.1%)		321 (53.7%)	277 (46.3%)		238 (39.7%)	360 (60.3%)		177 (29.6%)	421 (70.4%)	
No	110 (65.5%)	58 (34.5%)		122 (72.6%)	46 (27.4%)		94 (56.1%)	74 (44.0%)		83 (49.8%)	84 (50.2%)	
Spouse accepts me after SV†			0.03			<0.0001			0.0009			0.08
Yes	60 (33.4%)	120 (66.6%)		83 (46.1%)	97 (53.9%)		62 (34.8%)	117 (65.2%)		44 (24.6%)	135 (75.4%)	
No	61 (45.3%)	73 (54.7)%		95 (70.5%)	40 (29.5%)		72 (53.6%)	62 (46.4%)		45 (33.5%)	89 (66.5%)	
Family accepts me after SV‡			<0.0001			0.0004			0.0001			<0.0001
Yes	234 (42.3%)	318 (57.7%)		298 (54.0%)	254 (46.0%)		216 (39.1%)	336 (60.9%)		160 (29.1%)	392 (70.9%)	
No	129 (64.7%)	70 (35.3%)		137 (68.6%)	63 (31.4%)		109 (54.6%)	90 (45.4%)		92 (46.2%)	107 (53.8%)	
Community accepts me after SV§			<0.0001			<0.0001			<0.0001			<0.0001
Yes	145 (40.6%)	213 (59.5%)		190 (53.0%)	168 (47.0%)		138 (38.6%)	220 (61.4%)		88 (24.6%)	270 (75.4%)	
No	158 (62.5%)	95 (37.5%)		181 (71.7%)	71 (28.3%)		140 (55.7%)	112 (44.3%)		116 (45.8%)	137 (54.2%)	
Spouse, family or community accepts child after SV†			<0.0001			<0.0001			0.0021			<0.0001
Yes	249 (44.1%)	315 (55.9%)		302 (53.4%)	263 (46.6%)		226 (40.0%)	229 (60.0%)		162 (28.6%)	203 (71.4%)	
No	123 (61.3%)	78 (38.7%)		141 (70.3%)	60 (29.7%)		106 (52.5%)	95 (47.5%)		99 (49.1%)	102 (50.1%)	
Spouse accepts child†			0.05			0.001			0.01			0.02
Yes	47 (32.2%)	99 (67.8%)		68 (46.7%)	78 (53.3%)		52 (35.2%)	95 (64.8%)		34 (23.4%)	112 (76.6%)	
No	76 (42.8%)	102 (57.2%)		115 (64.8%)	63 (35.2%)		87 (49.2%)	90 (50.9%)		62 (35.1%)	115 (64.9%)	
Family accepts child‡			<0.0001			<0.0001			0.0002			<0.0001
Yes	220 (42.8%)	294 (57.2%)		269 (52.3%)	245 (47.7%)		199 (38.7%)	315 (61.3%)		138 (26.9%)	376 (73.1%)	
No	144 (60.3%)	95 (39.7%)		167 (70.0%)	72 (30.0%)		127 (53.4%)	111 (46.7%)		116 (48.7%)	122 (51.4%)	
Community accepts child§			<0.0001			<0.0001			<0.0001			<0.0001
Yes	133 (40.3%)	198 (59.7%)		171 (51.7%)	160 (48.3%)		127 (38.3%)	204 (61.7%)		77 (23.2%)	254 (76.8%)	
No	173 (58.0%)	125 (42.0%)		208 (69.8%)	90 (30.2%)		162 (54.4%)	136 (45.7%)		141 (47.3%)	157 (52.8%)	

*Suicidality includes women who reported prior suicide ideation or suicide attempt.

†Those who did not report a husband or who indicated their husband was not aware of the sexual violence were excluded from the analysis.

‡Those who indicated their family was not aware of the sexual violence were excluded from this analysis.

§Those who did not report that the community was aware of SV were excluded from the analysis.

SVRP, sexual violence-related pregnancy.

Crude and adjusted risk ratios demonstrate that stigma toward the woman from the community was associated with an increased risk for anxiety and suicidality. Crude and adjusted risk ratios demonstrate that stigma toward the child by the spouse, family or community was associated with an increased risk for all assessed mental health outcomes (table 4). After adjusting for important covariates, reported acceptance was no longer significantly associated with mental health outcomes (table 5).

**Table 4:** Unadjusted and adjusted* risk ratio (RR) and 95% CIs (95% CI) of characteristics of women raising children born from SVRPs

	Depression		PTSD		Anxiety		Suicidality†	
Variable	Unadjusted RR (95% CI)	Adjusted RR (95% CI)	Unadjusted RR (95% CI)	Adjusted RR (95% CI)	Unadjusted RR (95% CI)	Adjusted RR (95% CI)	Unadjusted RR (95% CI)	Adjusted RR (95% CI)
Has a spouse								
Yes	0.58 (0.48 to 0.71)	0.55 (0.45 to 0.69)	0.82 (0.72 to 0.95)	0.87 (0.75 to 1.00)	0.70 (0.57 to 0.85)	0.71 (0.57 to 0.88)	0.47 (0.35 to 0.62)	0.50 (0.37 to 0.67)
No	1.00	1.00	1.00	1.00	1.00	1.00	1.00	1.00
Education								
Any education	1.23 (1.06 to 1.43)	1.20 (1.02 to 1.41)	1.01 (0.90 to 1.14)	0.98 (0.87 to 1.12)	1.27 (1.07 to 1.50)	1.27 (1.06 to 1.53)	1.31 (1.07 to 1.61)	1.17 (0.94 to 1.45)
No education	1.00	1.00	1.00	1.00	1.00	1.00	1.00	1.00
Number of perpetrators								
Two or more	1.10 (0.94 to 1.30)	1.09 (0.89 to 1.33)	1.26 (1.04 to 1.52)	1.20 (1.01 to 1.42)	1.13 (0.97 to 1.30)	1.13 (0.91 to 1.41)	1.15 (1.02 to 1.29)	1.36 (1.02 to 1.82)
One	1.00	1.00	1.00	1.00	1.00	1.00	1.00	1.00
Pregnancy from captivity								
Yes	1.08 (0.86 to 1.35)	1.16 (0.93 to 1.5)	1.04 (0.87 to 1.25)	1.04 (0.86 to 1.26)	0.99 (0.78 to 1.26)	1.04 (0.81 to 1.34)	1.22 (0.89 to 1.68)	1.31 (0.95 to 1.80)
No	1.00	1.00	1.00	1.00	1.00	1.00	1.00	1.00
Stigma toward woman by community								
Yes	1.29 (1.10 to 1.52)	0.99 (0.83 to 1.18)	1.38 (1.22 to 1.57)	1.13 (0.99 to 1.30)	1.69 (1.43 to 2.01)	1.29 (1.07 to 1.56)	1.83 (1.47 to 2.28)	1.37 (1.07 to 1.74)
No	1.00	1.00	1.00	1.00	1.00	1.00	1.00	1.00
Stigma toward child by spouse, family or community								
Yes	1.35 (1.17 to 1.57)	1.32 (1.11 to 1.57)	1.52 (1.34 to 1.73)	1.45 (1.25 to 1.69)	1.62 (1.36 to 1.92)	1.39 (1.14 to 1.70)	1.59 (1.30 to 1.96)	1.31 (1.03 to 1.67)
No	1.00	1.00	1.00	1.00	1.00	1.00	1.00	1.00

*In addition to the above variables, adjusted for age of woman, age of child, if the community was aware of the assault and number of meals a day.

†Suicidality includes women who reported prior suicide ideation or suicide attempt.

SVRP, sexual violence-related pregnancy.

**Table 5:** Unadjusted and adjusted* risk ratio (RR) and 95% CIs (95% CI) of characteristics of women raising children born from SVRPs

	Depression		PTSD		Anxiety		Suicidality†	
Variable	Unadjusted RR (95% CI)	Adjusted RR (95% CI)	Unadjusted RR (95% CI)	Adjusted RR (95% CI)	Unadjusted RR (95% CI)	Adjusted RR (95% CI)	Unadjusted RR (95% CI)	Adjusted RR (95% CI)
Has a spouse								
Yes	0.58 (0.48 to 0.71)	0.58 (0.46 to 0.72)	0.82 (0.72 to 0.95)	0.87 (0.75 to 1.02)	0.70 (0.57 to 0.85)	0.70 (0.56 to 0.87)	0.47 (0.35 to 0.62)	0.52 (0.39 to 0.70)
No	1.00	1.00	1.00	1.00	1.00	1.00	1.00	1.00
Education								
Any education	1.23 (1.06 to 1.43)	1.22 (1.04 to 1.44)	1.01 (0.90 to 1.14)	1.02 (0.89 to 1.15)	1.27 (1.07 to 1.50)	1.32 (1.10 to 1.59)	1.31 (1.07 to 1.61)	1.19 (0.96 to 1.48)
No education	1.00	1.00	1.00	1.00	1.00	1.00	1.00	1.00
Number of perpetrators								
Two or more	1.10 (0.94 to 1.30)	1.06 (0.87 to 1.30)	1.26 (1.04 to 1.52)	1.17 (0.98–1.41)	1.13 (0.97 to 1.30)	1.10 (0.88 to 1.38)	1.15 (1.02 to 1.29)	1.32 (0.98 to 1.77)
One	1.00	1.00	1.00	1.00	1.00	1.00	1.00	1.00
Pregnancy from captivity								
Yes	1.08 (0.86 to 1.35)	1.16 (0.92 to 1.45)	1.04 (0.87 to 1.25)	1.06 (0.88 to 1.28)	0.99 (0.78 to 1.26)	1.05 (0.82 to 1.35)	1.22 (0.89 to 1.68)	1.31 (0.96 to 1.79)
No	1.00	1.00	1.00	1.00	1.00	1.00	1.00	1.00
Acceptance of woman by spouse, family, or community								
Yes	0.67 (0.58 to 0.77)	0.82 (0.67 to 1.00)	0.74 (0.66 to 0.83)	0.84 (0.71 to 1.00)	0.71 (0.60 to 0.84)	0.81 (0.64 to 1.02)	0.59 (0.49 to 0.72)	0.78 (0.59 to 1.04)
No	1.00	1.00	1.00	1.00	1.00	1.00	1.00	1.00
Acceptance of child by spouse, family or community								
Yes	0.72 (0.62 to 0.83)	0.93 (0.76 to 1.13)	0.76 (0.68 to 0.86)	0.90 (0.76 to 1.06)	0.76 (0.65 to 0.90)	0.96 (0.77 to 1.20)	0.58 (0.48 to 0.71)	0.75 (0.57 to 0.98)
No	1.00	1.00	1.00	1.00	1.00	1.00	1.00	1.00

*In addition to the above variables, adjusted for age of woman, age of child, if the community was aware of the assault and number of meals a day.

†Suicidality includes women who reported prior suicide ideation or suicide attempt.

SVRP, sexual violence-related pregnancy.

## Discussion

This is the first study, to our knowledge, to assess mental health outcomes among women raising children from SVRPs in eastern DRC. Among those surveyed, women raising children from SVRPs had high rates of depression, PTSD and anxiety based on symptom criteria and high rates of suicidality. The data suggest that stigma following sexual violence may exacerbate mental health disorders. Overall, the principal findings highlight the mental health needs of women raising children from SVRPs—a population that may not be traditionally included in sexual violence research or programming—and these data could be applied to inform targeted programming and services for this population.

In our study, stigmatisation of women raising children from SVRPs appears to play a role in mediating mental health outcomes. Women who reported stigma toward themselves or their children were more likely to meet symptom criteria for most mental health disorders. Other studies of sexual violence survivors have similarly reported that stigmatisation mediated certain mental health outcomes.^15–17^ As it is possible that women raising children from SVRPs face intensified stigma or different forms of stigma than survivors without SVRPs,^10^ further characterisation of the relationship between stigma and mental health in this context is needed. It is important to consider that stigma may both mediate mental health disorders and also be a consequence of mental health disorders. Participatory research could further explore local constructs of stigma and mental health to provide culture-specific and context-specific evidence on the relationship between stigma and mental health. Furthermore, given the evidence emerging from studies of adolescent sexual violence survivors,^15^ ^17^ ^31^ future research could aim to understand the experiences of adolescents with SVRPs.

Several important manifestations of stigma were described by participants. The finding that over 20% of women with SVRPs reported rape as a result of incurred stigma is concerning, and protection and prevention of further violence are paramount. While not assessed in our study, other studies have noted that stigma following sexual violence may be a barrier to seeking health services^32^ ^33^ and to seeking justice.^34^ Our quantitative questions on stigma may not have captured the complexity of stigma related to SVRPs, and future standardised or validated assessments that incorporate ecological frameworks^4^ and build on stigma-related research in other health fields^35^ ^36^ would be beneficial to advancing understanding of stigma following SVRPs. Specific domains of stigma have been identified by previous research on intimate partner violence, including cultural stigma, stigma internalisation and anticipated stigma,^33^ and there is growing research on important dimensions of stigma described among sexual violence survivors in DRC.^6^ ^9^ ^10^ ^37^

Although stigma is important to understand among sexual violence survivors, our findings also highlight that it should not be assumed that women raising children from SVRPs universally face stigma. Over one-third of women did not report stigma from the community. This difference in perceived stigma could be explained by the limitations of the instruments used to assess stigma, but there could also be other factors that mitigate the experience of stigma by the survivor and associated mental health outcomes. While the unadjusted data suggest that acceptance of the woman and acceptance of the child by the spouse, family and community may be a protective factor for mental health outcomes, the adjusted data suggest that acceptance may be more complex. Multiple underlying factors may determine or confound the concept of acceptance, as suggested by mixed methods research in DRC.^10^ Among potential protective factors for mental health disorders noted in our study, women who reported having a spouse at the time of the survey were less likely to meet symptom criteria for mental health disorders. A mixed-methods study of sexual violence survivors in DRC also found that husband abandonment and widowhood were risk factors for social rejection and that receiving support from husbands following sexual violence was protective.^5^ Rejection of sexual violence survivors in DRC has been associated with poorer mental health outcomes.^9^ Further research on individual, family and community level factors could inform programming and interventions for sexual violence survivors and women raising children from SVRPs.

While data regarding interventions for women with SVRPs and their children are lacking, other sexual violence studies in DRC emphasise that interventions need to not only address individual survivors’ needs, but also facilitate reintegration and healthy relationships with family and community.^9^ ^37^ ^38^ Support to couples and families following sexual violence may be important in preventing stigma and rejection and in promoting acceptance.^9^ ^10^ ^37^ ^38^ Our data also provide further evidence to support integrated person-focused and community-focused interventions.^39^ Community antistigma interventions could include educational interventions to address common misconceptions or aim to shift stigma from the survivors to the perpetrators of violence.^34^ Guidelines recommend including members of the stigmatised group in the design, delivery and evaluation of interventions^40^ and care must be taken not to increase stigma and risk as a result of the intervention.^41^

To date, the majority of sexual violence research has been focused on assessing maladaptive outcomes after trauma; however, it is important to balance this focus with further research on preventing mental health disorders following sexual violence, determining mediating factors for mental health disorders and on assessing adaptation, positive coping, and resilience factors. In conflict settings, there is evidence to support early treatment interventions for PTSD, such as Narrative Exposure Therapy.^42^ ^43^ A controlled trial in eastern DRC among sexual violence survivors found that group psychotherapy compared to individual therapy significantly reduced PTSD symptoms, minimised depression and improved functioning.^3^ The data suggest that stigma is an important factor in exacerbating mental health disorders, and future research comparing types of therapies and interventions could be tailored to prevent and address stigma following sexual violence. Reports on children born of war have emphasised the importance of providing psychosocial support to women raising children from SVRPS, helping families honour the child's existence and providing support to the mother, and providing options for women during and after SVRP (ie, family planning and adoption services).^12^ For women who become pregnant from sexual violence, strengthening reproductive options and mental health support for women during the pregnancy and following the delivery of the child should be part of comprehensive programming.^12^ ^44^

## Limitations

The study provides data on mental health outcomes, stigma, and acceptance among a population of women that has not been included in traditional research approaches. The results may not be generalisable to the population of eastern DRC; however, the sample was large, and in this study, RDS may approximate a random sample as previously described.^22^ Transportation and budgetary constraints only allowed for women living near Bukavu to participate. The majority of sexual violence incidents in this study resulted from a single armed group; thus, our findings may not apply to civilian and/or single perpetrator incidents. While RDS methodology is useful to study hard-to-reach populations, the use of RDS in this context may have only sampled women who experienced a similar type of sexual violence (ie, from a single armed group) and who were well networked around this type of sexual violence. Thus, RDS may not have captured all women with SVRPs in this region.^22^ We relied on self-reporting; however, with the screening questions, sensitive nature of SVRPs and minimal incentive, we are confident that only eligible participants were interviewed. The survey asked sensitive questions, and it is possible participants exaggerated or under-reported responses or that questions were misinterpreted due to translation. It is also possible that despite review by local partners and mental health professionals, our study instruments do not match local constructs of mental health outcomes, stigma and acceptance nor do they capture all domains of mental health outcomes. Further, mental health disorders were based on symptom criteria and diagnostic interviews were not conducted. Owing to regional insecurity, the study was terminated early; thus, potential participants with coupons may not have been interviewed. Despite the early study termination, over 750 women presented for interviews within a 4-week study period, and the majority of variables reached equilibrium prior to study termination.^22^

## Conclusion

In conclusion, the findings demonstrate high rates of depression, PTSD, anxiety and suicidality among sexual violence survivors raising children from SVRPs. The data around stigma and acceptance suggest that decreasing community stigma and fostering acceptance of the women and children may mediate mental health disorders among this population. Further research and dialogue on stigma toward and acceptance of women following sexual violence, and of children born from SVRPs, are needed to develop more targeted interventions at the individual, family and community levels.
